# Prevalence and Factors Associated with the Triple Burden of Malnutrition among Mother-Child Pairs in Sub-Saharan Africa

**DOI:** 10.3390/nu13062050

**Published:** 2021-06-15

**Authors:** Bright Opoku Ahinkorah, Iddrisu Amadu, Abdul-Aziz Seidu, Joshua Okyere, Eric Duku, John Elvis Hagan, Eugene Budu, Anita Gracious Archer, Sanni Yaya

**Affiliations:** 1 Faculty of Health, School of Public Health, University of Technology, Sydney, NSW 2007, Australia; brightahinkorah@gmail.com; 2 Africa Centre of Excellence in Coastal Resilience, University of Cape Coast, Cape Coast PMB TF0494, Ghana; iddrisu.amadu@stu.ucc.edu.gh (I.A.); eric.duku@stu.ucc.edu.gh (E.D.); 3 Department of Fisheries and Aquatic Sciences, School of Biological Sciences, College of Agriculture and Natural Sciences, University of Cape Coast, Cape Coast PMB TF0494, Ghana; 4 Department of Population and Health, College of Humanities and Legal Studies, University of Cape Coast, Cape Coast PMB TF0494, Ghana; abdul-aziz.seidu@stu.ucc.edu.gh (A.-A.S.); joshuaokyere54@gmail.com (J.O.); budueugene@gmail.com (E.B.); 5 College of Public Health, Medical and Veterinary Sciences, James Cook University, Townsville, QLD 4811, Australia; 6 Department of Estate Management, Takoradi Technical University, Takoradi P.O. Box 257, Ghana; 7 Department of Health, Physical Education and Recreation, University of Cape Coast, Cape Coast PMB TF0494, Ghana; 8 Neurocognition and Action-Biomechanics-Research Group, Faculty of Psychology and Sport Sciences, Bielefeld University, 1001 31 Bielefeld, Germany; 9 School of Nursing and Midwifery, University of Health and Allied Sciences, Ho PMB 31, Ghana; 2016garcher@uhas.edu.gh; 10 School of International Development and Global Studies, University of Ottawa, Ottawa, ON K1N 6N5, Canada; sanni.yaya@uOttawa.ca; 11 The George Institute for Global Health, Imperial College London, London W12 0BZ, UK

**Keywords:** global health, malnutrition, mother-child pairs, Sub-Saharan Africa, triple burden

## Abstract

Despite concerns about the coexistence of overnutrition, undernutrition and micronutrient deficiencies, which is compositely referred to as the triple burden of malnutrition (TBM), little is known about the phenomenon in sub-Saharan Africa (SSA). We, therefore, aimed to examine the prevalence and investigate the factors associated with TBM in SSA. This study uses cross-sectional survey data collected through the Demographic and Health Surveys (DHS) Program from 2010 to 2019. Data from 32 countries in SSA were used for the analysis. The prevalence of TBM were presented in tables and maps using percentages. The predictors of TBM were examined by fitting a negative log-log regression to the data. The results were then presented using adjusted odds ratios (aORs) at 95% Confidence Intervals (CIs). Out of the 169,394 children, 734 (1%) suffered from TBM. The highest proportion of children with TBM in the four geographic regions in SSA was found in western Africa (0.75%) and the lowest in central Africa (0.21%). Children aged 1 [aOR = 1.283; 95% CI = 1.215–1.355] and those aged 2 [aOR = 1.133; 95% CI = 1.067–1.204] were more likely to experience TBM compared to those aged 0. TBM was less likely to occur among female children compared to males [aOR = 0.859; 95% CI = 0.824–0.896]. Children whose perceived size at birth was average [aOR = 1.133; 95% CI = 1.076–1.193] and smaller than average [aOR = 1.278; 95% CI = 1.204–1.356] were more likely to suffer from TBM compared to those who were larger than average at birth. Children born to mothers with primary [aOR = 0.922; 95% CI = 0.865–0.984] and secondary [aOR = 0.829; 95% CI = 0.777–0.885] education were less likely to suffer from TBM compared to those born to mothers with no formal education. Children born to mothers who attended antenatal care (ANC) had lower odds of experiencing TBM compared to those born to mothers who did not attend ANC [aOR = 0.969; 95% CI = 0.887–0.998]. Children born to mothers who use clean household cooking fuel were less likely to experience TBM compared to children born to mothers who use unclean household cooking fuel [aOR = 0.724; 95% CI = 0.612–0.857]. Essentially, higher maternal education, ANC attendance and use of clean cooking fuel were protective factors against TBM, whereas higher child age, low size at birth and being a male child increased the risk of TBM. Given the regional variations in the prevalence and risk of TBM, region-specific interventions must be initiated to ensure the likelihood of those interventions being successful at reducing the risk of TBM. Countries in Western Africa in particular would have to strengthen their current policies and programmes on malnutrition to enhance their attainment of the SDGs.

## 1. Introduction

Globally, the world is operating on the Sustainable Development Goals (SDGs). Given the importance of promoting zero hunger and nutritional needs of the world’s population, SDG 2.2 envisions ending all forms of malnutrition by 2030 [1]. With this target in mind, there has been a significant decline in malnutrition across the globe [2]. For example, the global prevalence of stunting declined from 29.5% in 2005 to 22.9% in 2016 [3]. Nevertheless, there is still a substantial proportion of people worldwide who are malnourished [2].

Available evidence approximates that 155, and 52 million children under age five are stunted and wasted, respectively [3]. Moreover, the global prevalence of undernourished people is estimated to have increased from 777 million in 2015 to 815 million in 2016 [4], thus making malnutrition and its concomitant issues important public health concern [5]. In addition, malnutrition is reported to be most prevalent in South-East Asia [6] and sub-Saharan Africa [7].

It is important to note that malnutrition is not concerned only with the sufficiency of food but extends to having the appropriate micronutrients [8]. Moreover, evidence suggests that malnutrition coexists with other health events [9,10]. The coexistence of undernutrition along with overweight and obesity within individuals, households and populations and across the life course is what has been referred to as the double burden of malnutrition (DBM) [11]. However, in recent times, there have been concerns about the coexistence of overnutrition, undernutrition and micronutrient deficiencies, which is compositely referred to as the triple burden of malnutrition (TBM) [8].

TBM is a fairly new concept and has, therefore, not received enough scholarship in the current discourse on malnutrition among children under five [12]. Few studies have examined the prevalence and associated factors of TBM. Available evidence from elsewhere in Nepal [12] and India [2,8] have found factors such as maternal age, maternal educational status, caesarean section delivery, birth size of baby, household wealth quintile and place of residence to be significantly associated with TBM. Nonetheless, its prevalence and associated factors in the sub-Saharan African context have not been investigated, even though TBM poses a great threat to the health and wellbeing of children. This presents a gap in the current knowledge about TBM in sub-Saharan Africa (SSA) that ought to be filled. We, therefore, aimed to examine the prevalence and investigate the factors associated with TBM in the sub-Saharan African context. Knowing the prevalence and associated factors of TBM is a step in the right direction towards the attainment of SDG 2.2.

## 2. Materials and Methods

### 2.1. Study Design

**Figure 1:**
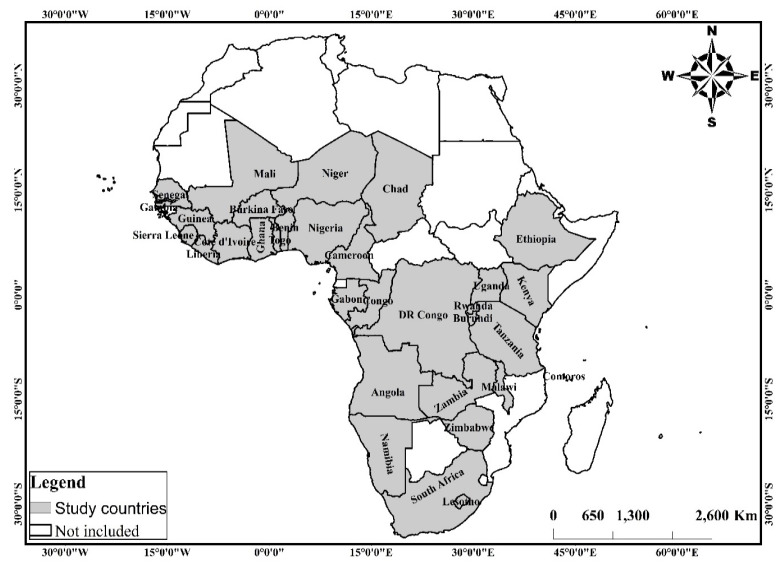
Spatial distribution of the study countries in Sub-Saharan Africa. Source: constructed based on shapefiles from https://tapiquen-sig.jimdofree.com/descargas-gratuitas/mundo/ (1 December 2020) with permission from Carlos Efrain Porto Tapiquen, 2021.

**Figure 2:**
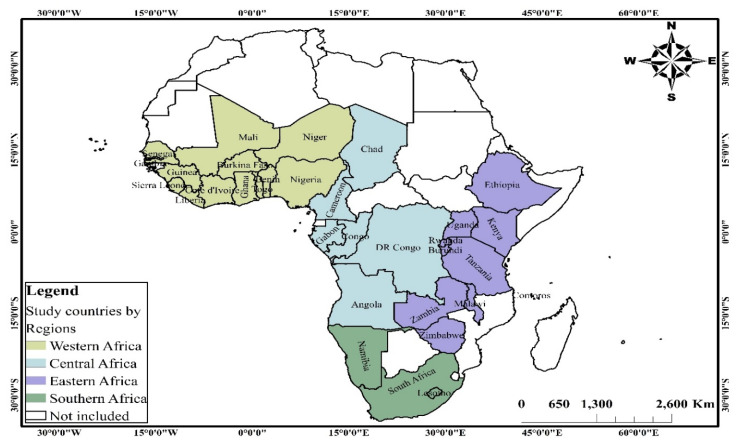
Spatial distribution of study countries by regions of Sub-Saharan Africa. Source: constructed based on shapefiles from https://tapiquen-sig.jimdofree.com/descargas-gratuitas/mundo/ (1 December 2020) with permission from Carlos Efrain Porto Tapiquen, 2021.

This study uses cross-sectional survey data collected through the Demographic and Health Surveys (DHS) Program from 2010 to 2019. The data of 32 countries in SSA (see Figure 1) in the geographic regions, western, eastern, central and southern Africa (see Figure 2), were obtained for analysis. For each geographic region in SSA, countries were considered based on the availability of data on (i) key anthropometrics and background characteristics including sex, height-for-age z-scores, weight-for-height z-scores, weight-for-age z-scores and anaemia level of children under the age of 5 years and their respective mothers; (ii) household characteristics including the background characteristics of household head and household’s access to basic services such as water, toilet facility and cooking fuel, among others.

### 2.2. Data Source, Sampling and Data Collection Procedure

The DHS Program since 1984 has gathered nationally representative data on important population, nutrition and other health indicators of women, men and children at the household level in over 90 low-to-middle-income countries around the world. The program employs standardised protocols and instruments in all its surveys to allow for inter-country comparisons. A two-stage stratified sampling technique involving the demarcation of enumeration areas (clusters) and household selection for interviews was done. Questionnaires are often translated into a country’s major local language, pre-tested and validated before implementation of the surveys. This study included 169,394 child-mother pairs who had complete data for all the variables of interest. We adhered to the strengthening the reporting of observational studies in epidemiology (STROBE) statement for developing this manuscript. The dataset can be accessed freely by download at: https://dhsprogram.com/data/available-datasets.cfm (22 March 2021).

### 2.3. Measurements

#### 2.3.1. Outcome Variable

The outcome variable TBM was derived from four child malnutrition indicators (stunting, wasting, underweight and anaemia status) and the body mass index (BMI) of their respective mothers. For parsimony and relevance to this study, anaemia levels were measured using four response categories (severe, moderate, mild and not anaemic), which were dichotomized into “anaemic” and “normal”, where anaemic was “severe”, “moderate” and “mild” were combined and coded as “1”, and not anaemic was labelled “normal” and coded “0”. Additionally, following previous studies [13], stunting, wasting, underweight and BMI of the mother were dichotomized and coded as 0 for “normal” and 1 for “stunted”, “wasting”, “underweight” and “obese/overweight”, respectively. Four combinations of these variables—Obese/overweight Mother and Anaemic Child (OM/AC), Obese/overweight Mother and Stunted Child (OM/SC), obese/overweight mother and wasted child (OM/WC), and obese/overweight mother and underweight child (OM/UC)—were made. Following Kumar et al. [2], the binary response variable TBM was measured using response categories “normal” and “TBM”, where the latter included obese/overweight mother with an undernourished child, i.e., children with stunting/wasting/underweight who were also anaemic.

#### 2.3.2. Independent Variables

The independent variables included in this study were considered based on literature and the availability of data. Previous studies [7,13,14,15,16] have documented several variables associated with child malnutrition spanning child, mother and household characteristics and contextual factors. The relevant variables on child characteristics considered include the age of the child in years (0, 1, 2, 3, 4); sex of child (male, female); birth order (1, 2, 3 and above); perceived birth size (larger than average, average, smaller than average, do not know). With regards to the mothers’ characteristics, the relevant variables include the age of mother in years (15–19, 20–24, 25–29, 30–34, 35–49, 40–44, 45–49); educational attainment (no formal, primary, secondary, higher); employment status (no, yes); antenatal care (ANC) visits (no, yes); postnatal care (PNC) visits (No, Yes). The household characteristics considered are the age of household head (“young adults” for those below 35 years, “middle-aged adults” for 35–55 years and “old-aged adults” for those aged 55 years and above [17]; sex of household head (male, female); household size (“small” for those with 1–5 members, “medium” for 6–10 members and “large” for more than 10 members (see [17,18]); wealth status (poor, middle, rich); access to electricity (no, yes); source of drinking water (improved, unimproved [17,18]); type of toilet facility (improved, unimproved [17,18]); and type of cooking fuel (unclean, clean [19,20]). Urbanicity (urban, rural) and geographic region (western Africa, eastern Africa, central Africa and southern Africa) were the contextual variables included in this study.

### 2.4. Data Analyses

All statistical analyses were performed using the Stata SE version 14.2 (StataCorp, College Station, TX, USA) software. Before analyses were conducted, the data were first declared as survey data using the Stata command “svyset” specifying the cluster, sample weighting and strata variables. This procedure was done to allow for robust estimation of effect sizes by preventing potential clustering and adjusting for oversampling and undersampling. Descriptive statistics (frequencies and percentages) were used to present the distribution of all variables of interest in tables. To enhance visualization and understanding of the study context, the data were then integrated into a geographic information system (GIS) environment and key variables presented in maps. The Chi-square test of independence was then used to assess the associations between the independent variables and the TBM. All independent variables associated with the TBM were tested for multicollinearity and there was no evidence of multicollinearity (see Table S1). The effects of these independent variables on the TBM were then examined by fitting a negative log-log regression to the data. A negative log-log generalized linear regression was deemed plausible considering the skewed distribution of the TBM to the non-affirmation (99%) [19,21,22]. The results were then presented using adjusted odds ratios (aORs) at 95% Confidence Intervals (CIs).

### 2.5. Ethical Approval

Ethical clearance for DHS reports is taken from the Ethics Committee of ORC Macro Inc. as well as the ethics boards of partner institutions (e.g., ministries of health) of the studied countries. The DHS protocols guarantee that ethical standards for the protection of respondents’ anonymity, privacy and confidentiality are adhered to. Inner City Fund International also ensures that the survey meets the United States Department of Health and Human Services’ regulations for the respect of human subjects. The study used secondary datasets; hence, no further ethical approval was required. The datasets can be accessed freely via download. Further information about the DHS data usage and ethical standards is available at http://goo.gl/ny8T6X (22 March 2021).

## 3. Results

### 3.1. Descriptive Analysis on TBM Across Four Geographic Regions in SSA

**Figure 3:**
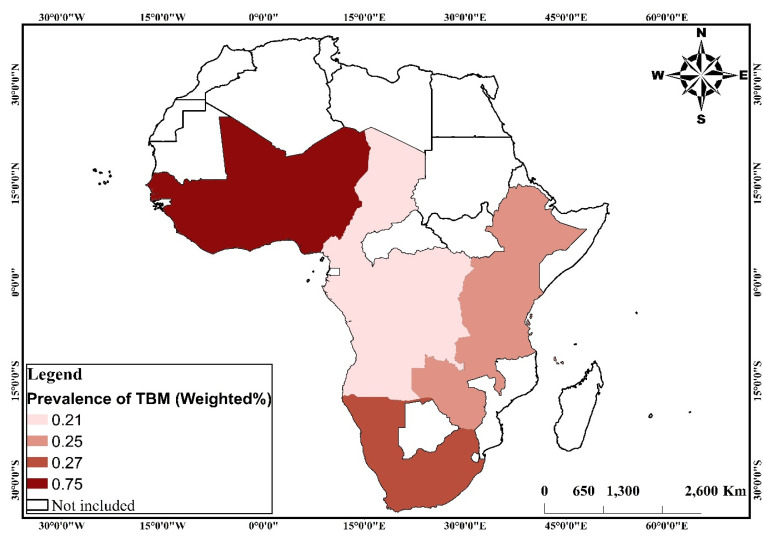
Maps showing the prevalence of triple burden of malnutrition in the four geographic regions in Sub-Saharan Africa. Source: constructed based on shapefiles from https://tapiquen-sig.jimdofree.com/descargas-gratuitas/mundo/ (1 December 2020) with permission from Carlos Efrain Porto Tapiquen, 2021. **TBM:** Triple burden of malnutrition.

The study included 169,394 children under five from 32 countries in SSA. Variations in sample size for the variables included in this study can be found in Table 1. The highest proportion of children with TBM in the four geographic regions in SSA was found in Western Africa (0.75%) and the lowest in Central Africa (0.21%) (Figure 3). There were variations in the prevalence of TBM across the child, mother, household and contextual factors. Factors that showed significant variations in TBM were the age of the child, sex of the child, birth order, perceived birth size, educational attainment, employment status and ANC. Other statistically significant factors were the age of the household head, sex of household head, household size, wealth status, access to electricity, source of drinking water, type of toilet facility, type of cooking fuel, urbanicity, and geographic region (Table 2).

### 3.2. Multivariate Analysis on the Predictors of TBM

Table 3 shows the results from the negative log-log regression analysis on the predictors of TBM. With the child factors, we found that children aged 1 [aOR = 1.283; 95% CI = 1.215–1.355] and those aged 2 [aOR = 1.133; 95% CI = 1.067–1.204] were more likely to experience TBM compared to those aged 0. TBM was less likely to occur among female children compared to males [aOR = 0.859; 95% CI = 0.824–0.896]. Children whose perceived size at birth was average [aOR = 1.133; 95% CI = 1.076–1.193] and smaller than average [aOR = 1.278; 95% CI = 1.204–1.356] were more likely to suffer from TBM compared to those who were larger than average at birth. In terms of the maternal factors, children born to mothers with primary [aOR = 0.922; 95% CI = 0.865–0.984] and secondary [aOR = 0.829; 95% CI = 0.777–0.885] education were less likely to suffer from TBM compared to those born to mothers with no formal education. Children born to mothers who attended ANC had lower odds of experiencing TBM compared to those born to mothers who did not attend ANC [aOR = 0.969; 95% CI = 0.887–0.998]. With the household factors, children born to mothers who use clean household cooking fuel were less likely to experience TBM compared to children born to mothers who use unclean household cooking fuel [aOR = 0.724; 95% CI = 0.612–0.857]. In terms of the contextual factors, children born to mothers who lived in eastern Africa [aOR = 0.837; 95% CI = 0.789–0.888] and central Africa [aOR = 0.822; 95% CI = 0.768–0.880] had lower odds of suffering from TBM compared to children born to mothers who lived in western Africa.

## 4. Discussion

This study examined the prevalence and factors associated with TBM in SSA. At the regional level, Western Africa recorded the highest prevalence of TBM, whereas Central Africa had the lowest prevalence. Additionally, our analysis indicates that children born to mothers who lived in Eastern Africa and Central Africa had lower odds of suffering from TBM compared to children born to mothers who lived in Western Africa. The result is consistent with an earlier study by Akombi et al. [14] that found a high prevalence of wasting and underweight in Western Africa compared to Central Africa. Plausibly, the high prevalence of TBM in Western Africa may be linked to the low rates of exclusive breastfeeding and vaccination for children when compared to Eastern Africa or Central Africa [23,24,25]. For example, Yourkavitch et al. [25] observed from their study that exclusive breastfeeding and vaccination for children were significantly low in Western Africa, and this is further exacerbated by the high levels of stunting in the region. Hence, this may explain the high prevalence of TBM observed with respect to Western Africa. Other studies attribute the high prevalence of TBM in Western Africa to the effects of rapid desertification on food access, availability and production, consequently leading to poor nutritional outcomes for children under five [17,26]. It is also worthy of note that policy differences, as well as other commitments towards addressing malnutrition, could account for sub-regional variations in the prevalence of TBM. For instance, [27] indicate that three out of 16 countries in Western Africa, two out of five in Southern Africa, four out of nine in Central Africa and seven out of 18 in Eastern Africa have multisectoral comprehensive nutrition plans.

Our study also shows that there is a combination of child-related, maternal-related, household and context level factors that were significantly associated with TBM in SSA. Concerning the child-related factors, the analysis showed that the age of the child, sex of the child, birth order and perceived birth size were significantly associated with TBM. Children older than age zero were more likely to experience TBM. This result is substantiated by a recent study from Nepal [2] that found higher child age to be significantly associated with higher risks of TBM. Extant studies have shown that childhood anaemia, faltering growth, stunting, wasting and underweight are more profound in children after age zero [28,29], hence explaining the higher odds of TBM among children aged 1 or 2 as compared to those aged zero.

Consistent with a previous study by Kumar et al. [2], we found that TBM was more likely to occur among children whose perceived size at birth was average or smaller than average. This finding is not surprising given that stunting, underweight, wasting and childhood anaemia are significantly associated with low size at birth [15,30]. In addition, children with low size at birth tend to lack sufficient amounts of nutrients necessary to promote their growth [2]. Hence, such children are more likely to suffer TBM. Contrary to a related study in Nepal [12] that found no significant association between sex of a child and TBM, our study revealed that there was a significant association, with female children having a reduced risk of TBM compared to the males. This can be explained from the perspective of the evolutionary theory that posits that the differences in the genetic composition of males and females (also known as selective male mortality) account for the higher risk of TBM among males compared to female children [31,32,33].

Maternal educational attainment and ANC attendance were the only maternal factors that were significantly associated with TBM among children under five. Compared to children born to mothers with no formal education, those born to mothers with primary and secondary education were less likely to suffer from TBM. Likewise, the odds of TBM were significantly lower among children born to mothers who attended ANC compared to those born to mothers who did not attend ANC. What these findings imply is that maternal education and ANC are strong protective factors to control TBM. The results are congruent to reports from elsewhere [12]. A possible justification for this finding could be that, during ANC, mothers are provided with micronutrient supplementation and fortification such as the folic acid that promotes the health of the mother while meeting the nutritional needs of the child [34], hence reducing the risk of TBM. Furthermore, higher maternal education increases women’s decision-making capacity and also empowers mothers by equipping them with the relevant knowledge concerning the nutritional needs of children [35].

Concerning the household factors, cooking fuel was the only factor that was significantly associated with TBM. Children born to mothers who use clean household cooking fuel were less likely to experience TBM compared to children born to mothers who use unclean household cooking fuel. Similar findings have been reported by Amadu et al. [13] that showed that unclean cooking fuel exacerbated the risk of stunting, wasting and underweight. Available evidence suggests that the use of unclean cooking fuel worsens household air pollution, which can operate through other pathways to impair growth in children and subsequently lead to TBM [36,37]. Therefore, the current findings emphasise the need for sub-Saharan African countries to invest in clean cooking fuel.

### 4.1. Strengths and Limitations

The strength of this study lies in the robust analytical and statistical methods used. This enhances the trustworthiness of our findings. Additionally, we provide a detailed methodological procedure, hence making our study replicable. The use of a nationally representative dataset makes the findings of this study generalisable to all children under five in SSA. Our findings make a significant contribution to knowledge by being the first to investigate the factors associated with TBM within the sub-Saharan African context. As such, it will set the pace for more research to be done in this regard. Nevertheless, our study has some noteworthy limitations; therefore, an interpretation of the findings should be done with caution.

Given that the DHS dataset employed a cross-sectional design, we cannot establish causality between the various factors (i.e., child-related, maternal, household and contextual factors) and TBM. Additionally, since questions on TBM were self-reported, there could be some recall bias, which is beyond the control of the team. The pooling of the data may be affected by heterogeneity across regions.

### 4.2. Practical Implications

Not only does our findings contribute to bridging the knowledge gap on TBM in SSA, it also has considerable implications for policy and practice. From our study, it is evident that modifiable maternal and household factors such as maternal education, ANC attendance and use of clean cooking fuel significantly lower the risk of TBM. Therefore, if the sub-region is to attain SDG 2.2 (ending all forms of malnutrition by 2030) and SDG 3.2, then the individual countries should strengthen policies on female education. Additionally, the current findings highlight the essentiality of investing in clean cooking fuel (e.g., electricity, gas, ethanol, solar) for sub-Saharan African countries. Again, the contextual variation indicates that Western Africa must put in serious effort to combat TBM. Free maternal healthcare policies, as seen in the case of Ghana, should be exemplified in other western African countries to achieve the desired reduction in TBM.

## 5. Conclusions

This study aimed to examine the prevalence and factors associated with TBM in SSA. We conclude that the prevalence of TBM is higher in Western Africa than in any other region. Additionally, the study revealed that a combination of child-related, maternal, household and contextual factors were associated with TBM. Essentially, higher maternal education, ANC attendance and use of clean cooking fuel were protective factors against TBM, whereas higher child age, low size at birth and being a male child increased the risk of TBM. Given the regional variations in the prevalence and risk of TBM, region-specific interventions must be initiated to ensure the likelihood of those interventions being successful at reducing the risk of TBM. Countries in Western Africa in particular would have to strengthen their current policies and programmes on malnutrition and to enhance their attainment of the SDGs.

## Figures and Tables

**Table 1:** Distributions of child, mother, household and contextual variables.

Variable	Weighted *n*	(%)	Variable	Weighted *n*	(%)
OM/WC			Employment status		
Normal	166,058	98	No	59,817	37
Wasting	3335	2	Yes	100,628	63
OM/SC			ANC		
Normal	156,387	92	No	12,262	10
Stunting	13,007	8	Yes	106,281	90
OM/UC			PNC		
Normal	162,158	96	No	63,838	59
Underweight	7236	4	Yes	45,140	41
OM/AC			**Household characteristics**	
Normal	148,790	88	Age of household head	
Anemic	20,604	12	Young adults	75,105	44
TBM			Middle-aged adults	73,311	43
Normal	168,660	99	Old-aged adults	20,975	12
OM/SC/WC/UC and AC	734	1	Sex of household head	
**Child characteristics**			Male	136,212	80
Age of child			Female	33,182	20
0	34,564	20	Household size		
1	35,793	21	Small	69,944	41
2	33,487	20	Medium	78,415	46
3	33,693	20	Large	21,035	12
4	31,856	19	Wealth status		
Sex of child			Poor	75,261	44
Male	85,523	50	Middle	33,996	20
Female	83,871	50	Rich	60,136	36
Birth order			Access to electricity		
1	35,347	21	No	119,345	70
2	81,981	48	Yes	50,032	30
3 and above	52,065	31	Source of drinking water	
Perceived birth size			Improved	109,141	64
Larger than average	54,853	34	Unimproved	60,232	36
Average	78,374	49	Type of toilet facility		
Smaller than average	24,950	16	Improved	72,110	43
Do not know	2314	1	Unimproved	97,246	57
**Mother characteristics**			Type of cooking fuel		
Age of mother			Unclean	154,729	91
15–19	9383	6	Clean	14,648	9
20–24	36,523	22	**Contextual**		
25–29	47,310	28	Urbanicity		
30–34	36,577	22	Urban	52,513	31
35–49	25,071	15	Rural	116,881	69
40–44	11,200	7	Geographic region		
45–49	3330	2	Western Africa	63,978	38
Educational attainment			Eastern Africa	69,144	41
No formal	67,012	40	Central Africa	32,899	19
Primary	58,079	34	Southern Africa	3373	2
Secondary	38,671	23	*n*	169,394	
Higher	5633	3			

NB: Obese/overweight Mother and Anaemic Child = OM/AC; Obese/overweight Mother and Stunted Child = OM/SC, obese/overweight mother and wasted child = OM/WC; obese/overweight mother and underweight child = OM/UC; antenatal care = ANC; postnatal care = PNC.

**Table 2:** Associations between child, mother, household and contextual factors and TBM.

Variable	TBM (Weighted %)	95% CIs	*p*-Value	Variable	TBM (Weighted %)	95% CIs	*p*-Value
**Child characteristics**				**Household characteristics**			
Age of child				Age of household head			
0	0.26	0.21–0.30	<0.001	Young adults	0.37	0.32–0.41	0.001
1	0.88	0.79–0.98		Middle-aged adults	0.50	0.45–0.55	
2	0.40	0.34–0.48		Old-aged adults	0.43	0.37–0.55	
3	0.29	0.23–0.35		Sex of household head			0.008
4	0.30	0.24–0.37		Male	0.46	0.43–0.50	
Sex of child				Female	0.31	0.26–0.38	
Male	0.56	0.51–0.61	<0.001	Household size			
Female	0.31	0.27–0.34		Small	0.36	0.32–0.41	<0.001
Birth order				Medium	0.41	0.37–0.46	
1	0.31	0.25–0.37	<0.001	Large	0.74	0.63–0.86	
2	0.39	0.34–0.43		Wealth status			
3 and above	0.59	0.53–0.66		Poor	0.56	0.51–0.62	<0.001
Perceived birth size				Middle	0.42	0.36–0.50	
Larger than average	0.27	0.22–0.32	<0.001	Rich	0.28	0.24–0.32	
Average	0.47	0.42–0.52		Access to electricity			
Smaller than average	0.83	0.72–0.95		No	0.48	0.44–0.53	<0.001
Do not know	0.51	0.28–0.90		Yes	0.31	0.27–0.37	
**Mother characteristics**				Source of drinking water			
Age of mother				Improved	0.42	0.38–0.46	0.038
15–19	0.40	0.28–0.54	0.284	Unimproved	0.46	0.41–0.52	
20–24	0.33	0.28–0.40		Type of toilet facility			
25–29	0.42	0.36–0.48		Improved	0.28	0.24–0.32	<0.001
30–34	0.46	0.39–0.53		Unimproved	0.55	0.50–0.59	
35–49	0.55	0.46–0.70		Type of cooking fuel			
40–44	0.45	0.33–0.59		Unclean	0.47	0.43–0.50	<0.001
45–49	0.69	0.44–10.3		Clean	0.08	0.04–0.10	
Educational attainment				**Contextual**			
No formal	0.75	0.69–0.82	<0.001	Urbanicity			
Primary	0.28	0.24–0.33		Urban	0.26	0.22–0.31	<0.001
Secondary	0.15	0.11–0.19		Rural	0.51	0.47–0.55	
Higher	0.20	0.10–0.35		Geographic region			
Employment status				Western Africa	0.75	0.69–0.82	<0.001
No	0.51	0.45–0.57	<0.001	Eastern Africa	0.25	0.22–0.29	
Yes	0.43	0.39–0.47		Central Africa	0.21	0.16–0.26	
ANC				Southern Africa	0.27	0.12–0.51	
No	0.91	0.75–10.9	<0.001				
Yes	0.42	0.38–0.46					
PNC							
No	0.54	0.48–0.60	0.113				
Yes	0.43	0.37–0.50					

**Table 3:** Negative log-log regression showing relationships between TBM and predictor variables.

Variable	aOR	Robust SE	*p*-Value	95% CIs	
**Child characteristics**					
Age of child (Ref: 0)					
1	1.283	0.036	<0.001	1.215	1.355
2	1.133	0.035	<0.001	1.067	1.204
3	0.996	0.043	0.928	0.916	1.084
4	1.013	0.051	0.792	0.918	1.118
Sex of child (Ref: male)					
Female	0.859	0.019	<0.001	0.824	0.896
Perceived birth size (Ref: larger than average)					
Average	1.133	0.030	<0.001	1.076	1.193
Smaller than average	1.278	0.039	<0.001	1.204	1.356
Do not know	1.180	0.117	0.094	0.972	1.432
Birth order (Ref: 0)					
2	1.031	0.032	0.329	0.970	1.096
3 and above	1.062	0.037	0.084	0.992	1.136
**Mother characteristics**					
Educational attainment (Ref: no formal)					
Primary	0.922	0.030	0.014	0.865	0.984
Secondary	0.829	0.028	<0.001	0.777	0.885
Higher	0.955	0.086	0.611	0.800	1.140
Employment status (Ref: no)					
Yes	0.969	0.022	0.155	0.927	1.012
ANC (Ref: no)					
Yes	0.941	0.028	0.043	0.887	0.998
**Household characteristics**					
Age of household head (Ref: young adult)					
Middle-aged adults	1.020	0.026	0.451	0.969	1.073
Old-aged adults	0.981	0.032	0.552	0.920	1.046
Sex of household head (Ref: male)					
Female	0.973	0.029	0.362	0.917	1.032
Household size (Ref: small)					
Medium	0.980	0.026	0.453	0.930	1.033
Large	1.059	0.034	0.078	0.994	1.128
Wealth status (Ref: poor)					
Middle	0.978	0.026	0.415	0.928	1.031
Rich	0.958	0.030	0.170	0.901	1.018
Source of drinking water (Ref: improved)					
Unimproved	0.985	0.021	0.472	0.945	1.027
Type of toilet facility (Ref: improved)					
Unimproved	1.052	0.030	0.077	0.994	1.113
Type of Cooking fuel (Ref: unclean)					
Clean	0.724	0.062	<0.001	0.612	0.857
Access to electricity (Ref: no)					
Yes	0.992	0.031	0.785	0.932	1.054
**Contextual factors**					
Urbanicity (Ref: urban)					
Rural	1.035	0.033	0.283	0.972	1.102
Geographic region (Ref: western Africa)					
Eastern Africa	0.837	0.025	<0.001	0.789	0.888
Central Africa	0.822	0.028	<0.001	0.768	0.880
Southern Africa	0.859	0.080	0.103	0.715	1.031

## Data Availability

The dataset is available on the following website: http://goo.gl/ny8T6X (22 March 2021).

## Supplementary Materials

The following are available online at https://www.mdpi.com/article/10.3390/nu13062050/s1, Table S1: Test of Multicollinearity between All Independent Variables and TBM.

## Abbreviations

SSA	Sub-Saharan Africa
TBM	Triple burden of malnutrition
DBM	Double burden of malnutrition
ANC	Antenatal care visits
PNC	Postnatal care visits
SDGs	Sustainable Development Goals
DHS	Demographic and Health Surveys
OM/AC	Obese/overweight mother and anaemic child
OM/SC	Obese/overweight mother and stunted child
OM/WC	Obese/overweight mother and wasted child
OM/UC	Obese/overweight mother and underweight child
STROBE	Strengthening the reporting of observational studies in epidemiology
aOR	Adjusted odds ratios
OR	Odds ratio
CI	Confidence intervals
