# Supplementary material for: Prevalence and Factors Associated with the Triple Burden of Malnutrition among Mother-Child Pairs in Sub-Saharan Africa
Source: Nutrients. 2021 Jun 15;13(6):2050. doi: 10.3390/nu13062050 (PMC8232587; doi:10.3390/nu13062050)
Supplement: Supplementary file 1 [file nutrients-13-02050-s001.zip › nutrients-1242917-supplementary.pdf]

**Variable**	**VIF**	**SQRT VIF**	**Tolerance**	**R-Squared**
Age of child	1.03	1.01	0.971	0.029
Sex of child	1.00	1.00	0.996	0.004
Perceived birth size	1.01	1.01	0.986	0.014
Birth order	1.22	1.11	0.818	0.182
Educational attainment	1.48	1.22	0.677	0.323
Employment status	1.05	1.02	0.956	0.044
ANC	1.11	1.05	0.903	0.097
Age of household head	1.26	1.12	0.795	0.205
Sex of household head	1.04	1.02	0.962	0.038
Household size	1.36	1.17	0.735	0.266
Wealth status	1.77	1.33	0.565	0.435
Source of drinking water	1.09	1.04	0.918	0.082
Type of toilet facility	1.29	1.14	0.774	0.227
Type of cooking fuel	1.33	1.15	0.754	0.246
Access to electricity	1.72	1.31	0.582	0.418
Urbanicity	1.58	1.26	0.632	0.368
Geographic region	1.17	1.08	0.852	0.148
Mean	1.27			
